# Ovarian mucinous adenocarcinoma with functioning stroma in postmenopausal women: aromatase and SF-1 expressions

**DOI:** 10.1186/s13048-015-0202-y

**Published:** 2015-11-14

**Authors:** Yuka Hattori, Shizuka Yamada, Makoto Yamamoto, Makoto Orisaka, Tetsuya Mizutani, Yoshio Yoshida

**Affiliations:** Department of Obstetrics and Gynecology, Sugita Genpaku Memorial Obama Municipal Hospital, Fukui, Japan; Department of Obstetrics and Gynecology, University of Fukui, Fukui, Japan; Department of Biochemistry, Faculty of Medical Sciences, University of Fukui, Fukui, Japan

**Keywords:** Epithelial ovarian tumors, Functioning stroma, Estradiol, Aromatase, Steroidogenic factor-1

## Abstract

**Background:**

A high serum estradiol (E2) level is occasionally detected in postmenopausal women with common epithelial ovarian tumors with functioning stroma. It has been proven that functioning stroma has the capacity to convert androgens to estrogens. However, the mechanism of the initiation and development of functioning stroma remains unclear.

**Case Presentation:**

We present two cases of elevated E2 levels in elderly women with ovarian mucinous adenocarcinomas that contained functioning stroma. Immunohistochemical evaluation revealed high expression levels of aromatase and steroidogenic factor-1 (SF-1), which is considered to be a master regulator of steroidogenesis, in their ovarian stroma.

**Conclusions:**

These cases suggest that overexpression of SF-1 may promote estrogen biosynthesis through regulation of P450 aromatase expression in ovarian tumors with functioning stroma; this in turn induces high serum E2 levels in postmenopausal women with common epithelial ovarian tumors.

## Background

It is known that elevated serum levels of steroid hormones including estradiol (E_2_) are present in postmenopausal women with common epithelial ovarian tumors [1–4]. Such ovarian tumor stroma exhibiting lutein-like or theca-like cells, or both, is frequently detected with morphological studies and has been termed “ovarian tumors with functioning stroma” [1]. Recently, from a pathogenic perspective, it has been reported that P450 aromatase, which converts androgens to estrogens, was exclusively expressed in stromal cells, although not all serum estrogens are produced in functioning stroma [5]. However, the mechanism of the initiation and development of ovarian tumors with functioning stroma and the significance of the expression of P450 aromatase in functioning stroma are still unclear.

We briefly describe the presence of elevated E_2_ levels in postmenopausal women with ovarian mucinous adenocarcinoma; the tumors showed high expression levels of aromatase and steroidogenic factor-1 (SF-1), which is a critical regulator of reproduction that regulates the transcription of key genes involved in sexual development and reproduction [6]. Since this factor induces the differentiation of mesenchymal stem cells into steroidogenic cells, it is considered a master regulator of steroidogenesis [7].

## Patients and methods

### Patients

A preliminary retrospective analysis was conducted involving postmenopausal women with ovarian mucinous adenocarcinoma in whom serum E_2_ concentrations were measured prior to undergoing gynecologic surgery because of estrogen-related manifestations at the Department of Obstetrics and Gynecology at Sugita Genpaku Memorial Obama Municipal Hospital and University of Fukui from June 2015 to September 2015. The two patients were selected for this study because of their postmenopausal status, defined as the absence of menses for more than 10 years in this study, and they had elevated serum E_2_ concentrations. The normal serum E2 concentration after menopause is reported to be less than approximately 20 pg/ml [8], but these two patients had ovarian mucinous adenocarcinomas with morphologically functional stroma. After informed consent was obtained from each patient, for case 1, comparisons were made of the expressions of P450 aromatase and SF-1 by immunohistochemical analysis, and for case 2, comparisons were made of expressions of P450 aromatase and SF-1 by reverse transcription-quantitative real-time PCR (RT-qPCR) evaluation.

### Immunohistochemical analysis

All tissues were fixed in 4 % formalin and embedded in paraffin. Standard hematoxylin and eosin (H&E)-stained sections (5 μm) were made for microscopic histology evaluation. These tissues were immunohistochemically stained using the avidin-biotin-peroxidase complex technique with an LSAB kit (Dako, Glostrup, Denmark) [9]. Immunohistochemical staining was performed using primary antibodies to the following proteins, diluted in PBS: P450 aromatase (anti-cytochrome P450 2E1 antibody ab 28146, mouse monoclonal, 1:100, Abcam, Tokyo, Japan); and SF-1 (clone N1665, mouse monoclonal, 1:100, Perseus Proteomics, Tokyo, Japan). For the staining procedure, the DAKO Envision HRP mouse kit (Dako) with DAB as the detection method was used. Sections from human colon and breast cancers were used as positive controls, and, for negative controls, incubation with the primary antibody was omitted.

### RT-qPCR

We examined gene expressions of *SF-1* and *CYP19A1,* which encodes aromatase, in case 2 with ovarian mucinous adenocarcinoma, as well as in KGN cells, a tumor cell line derived from human ovarian granulosa cells [10] and uterine leiomyosarcoma. KGN cells have often been used as a positive control for *SF-1* and *CYP19A1* expressions, and the cells have aromatase activityto produce estrogen in the presence of its substrate (androstenedione). Leiomyosarcoma specimens were used as negative controls. RT-qPCR was performed as described previously [9]. Primers used for RT-qPCR were as follows: SF-1 (forward), 5′- CATCCTCTTCAGCCTGGATTTG-3′ and SF-1 (reverse), 5′-CCTTCTCCTGAGCGTCTTTCAC-3′; CYP19A1 (forward), 5′- TAGCAGAGAAACGTGGTGAC -3′ and CYP19A1 (reverse), 5′- GAGACAGACATGGTGTCAGG -3′; ACTB (forward), 5′- GGACTTCGAGCAAGAGATGG -3′ and ACTB (reverse), 5′- AAGGAAGGCTGGAAGAGTGC -3′.

## Results

**Fig. 1:**
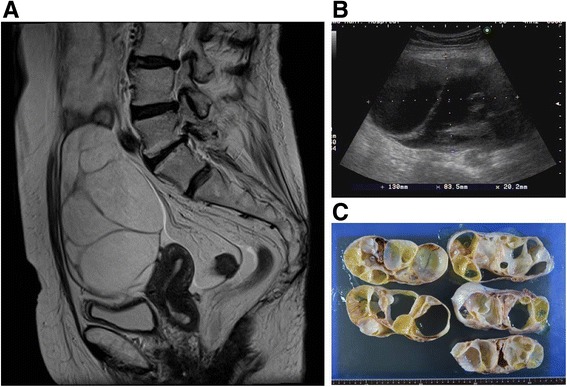
**a** Sagittal T2-weighted MRI shows a multilocular cystic mass, which is clearly delineated in the pelvis (maximum diameter approximately 20 cm). The uterus measures 80 mm in height, 55 mm in body length, and 6 mm in cavity thickness. **b** An ultrasound demonstrates the pelvic mass (diameter approximately 13 cm) containing a multilocular cyst without mural nodules. **c** Gross appearance, the tumor measures 19.0 cm x 14.0 cm x 8.5 cm. The inner surface of the cyst is mostly smooth, with areas of irregular thickening of the cyst wall, including yellowish mucoid substance

Case 1: An 83-year-old woman, a retired farmer, presented with a chief complaint of repeated postmenopausal bleeding and an increased feeling of abdominal distension. She had presented two years earlier with vaginal bleeding, and endometrial cytology had shown no abnormal findings. Her medical history included anxiety disorder and hyperlipidemia. She was a non-smoker. Physical examination revealed an obese woman with abdominal fullness. She had no palpable lymph nodes. Pelvic examination revealed vaginal mucosa that was young for her age and a slightly eroded vaginal fornix, with a uterus that was the size of a hen’s egg. Her serum E_2_ concentration was 80 pg/ml. Ultrasound examination revealed a multilocular cystic tumor without mural nodules. Magnetic resonance imaging (MRI) showed a multilocular cystic mass in the pelvis (maximum diameter approximately 20 cm). The wall of the mass was clearly delineated, and there was no evidence of any internal excrescences. The uterus was not atrophic (Fig. 1a, b).

**Fig. 2:**
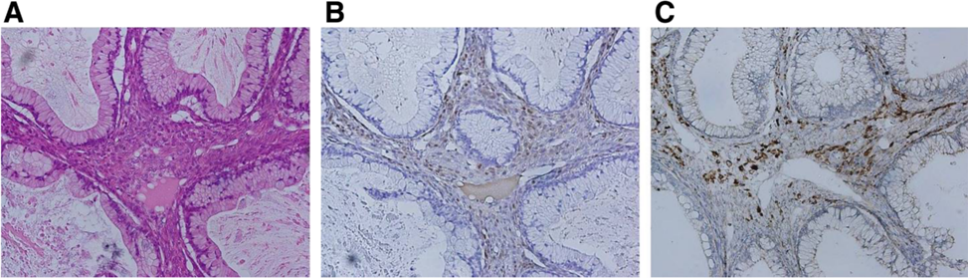
Histopathological features of the tumor with functioning stroma (H&E; 400×). Lutein-like cells contain abundant eosinophilic cytoplasm and round regular nuclei (**a**) On immunohistochemistry for SF-1 (**b**) and P450 aromatase (**c**), stromal cells are immunoreactive, but epithelial cells are not

Laparotomy revealed a large left adnexal tumor with an intact capsule. On gross examination, the tumor measured 19.0 cm x 14.0 cm x 8.5 cm. The inner surface of the cyst was mostly smooth, with areas of irregular thickening of the cyst wall (Fig. 1c). Microscopically, the tumor consisted primarily of a mucinous cystadenoma (Fig. 2a). In addition, there were 2- to 3-cell layer stratifications with nuclear atypia in some areas and minimal invasion. The final pathologic diagnosis was a mucinous adenocarcinoma. After surgery, her serum E_2_ level decreased to a normal postmenopausal level.

The tumor stroma contained lutein- and theca-like cells, so-called “ovarian tumors with functioning stroma”. The specimen was examined immunohistochemically for SF-1 and P450 aromatase. On immunohistochemistry for SF-1, stromal cells were immunoreactive for the enzyme, but epithelial cells were not. SF-1-positive stromal cells beneath the epithelial cells were somewhat aggregated (Fig. 2b). On immunohistochemistry for P450 aromatase, stromal cells were immunoreactive for the enzyme, but epithelial cells were not. Aromatase-positive stromal cells were somewhat aggregated in this tumor (Fig. 2c).

**Fig. 3:**
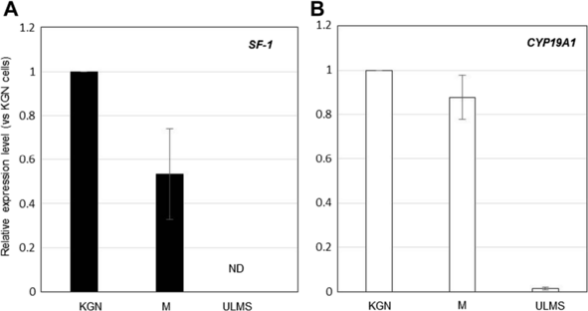
Expression levels of *SF-1* (**a**) and *CYP19A1* (**b**) in KGN cells, a tumor cell line derived from human ovarian granulosa cells, a mucinous adenocarcinoma (M), and a uterine leiomyosarcoma (ULMS). Six pieces were randomly collected from each specimen. The gene expressions of *SF-1* and *CYP19A1* were measured by quantitative real-time PCR. The mRNA levels were normalized against *ACTB*. Data are the means ± SEM (*n =* 6). ND, Not detected

Case 2: A 59-year-old woman, an office worker, presented with a chief complaint of hematuria. She noticed blood in her urine after she had fallen. She underwent detailed examination at the urology department, and an ovarian tumor was found by chance. She was referred to our department. She had been otherwise healthy. Physical examination revealed a slightly obese woman with abdominal fullness. She had no palpable lymph nodes. On pelvic examination, there was a whitish discharge, and the vaginal mucosa was young for her age, with a slightly eroded vaginal fornix. Ultrasonography and MRI revealed a multilocular cystic tumor (maximum diameter approximately 7 cm) with mural nodules in her pelvis, which highly suggested malignant ovarian tumors. Her serum E_2_ concentration was 28 pg/ml. She underwent laparotomy, and the tumor originated from the right ovary. Histopathological examination revealed that the tumor consisted primarily of a mucinous adenocarcinoma with functioning stroma. After surgery, her serum E_2_ level decreased to a normal postmenopausal level. RNA was extracted from frozen specimens of this tumor. To assess the gene expression levels of *SF-1* and *CYP19A1* in the tumor, RT-qPCR was performed. Both *SF-1* and *CYP19A1* genes were expressed in the tumor, and expression levels of these genes were markedly higher in the tumor than in uterine leiomyosarcoma (Fig. 3). These results suggest that SF-1 could work as a transcriptional activator for *CYP19A1*, which in turn leads to the production of estrogen in the tumor.

## Case Presentation

We present two cases of elevated E2 levels in elderly women with ovarian mucinous adenocarcinomas that contained functioning stroma. Immunohistochemical evaluation revealed high expression levels of aromatase and steroidogenic factor-1 (SF-1), which is considered to be a master regulator of steroidogenesis, in their ovarian stroma.

## Discussion

Hormonally active ovarian tumors, such as granulosa cell tumors, thecomas, and Sertoli-Leydig cell tumors, are classified as sex cord-stromal or steroid cell tumors. In several reports, elevated serum levels of steroid hormones including E_2_ were noted in postmenopausal women with common epithelial ovarian tumors. Both malignant and benign ovarian tumors produce E_2_. Several studies have reported relatively high peripheral E_2_ (>20 pg/ml) concentrations in about 50 % of postmenopausal women with ovarian epithelial tumors, compared to the levels observed in postmenopausal women without ovarian tumors [1–4]. Among them, the E_2_ released from ovarian tumors has biological effects on bone metabolism and increases the incidence of abnormal vaginal bleeding or abnormally thick endometrium [8].

In these tumors, the stroma produces steroid hormones, whereas the tumor cells do not. Morphologically, such ovarian tumor stroma has lutein- and theca-like cells (both are frequently detected), and they have been termed “ovarian tumors with functioning stroma” [1]. In such tumors, it has been unclear whether the tumor stroma itself could produce estrogen. Recently, Kato et al. reported that, on immunohistochemistry, P450 aromatase was exclusively expressed in the functioning stromal cells showing an increase in serum E_2_. However, there was no correlation between the immunoreactivity for P450 aromatase and the serum level of E_2_. They concluded that ovarian tumors with functioning stroma, regardless of cell morphology, have a capacity for converting androgens (mainly of adrenal origin) to estrogens, but this conversion usually takes place in peripheral tissues [5].

In case 1, immunoreactivity for P450 aromatase was detected in the functioning stromal cells. In addition, the preoperative serum level of E_2_ was high, and the postoperative serum level of E_2_ was not detectable. These findings may indicate a relatively high level of E_2_ derived from these ovarian tumors themselves. Thus, the next question is what is the mechanism of initiation and development of ovarian tumors with functioning stroma through the expression of p450 aromatase.

It has been shown that CYP19A1 expression is activated by SF-1 through its direct binding to the ovarian-specific promoter (pII) [11]. In fact, a chromatin immunoprecipitation study indicated that SF-1 bound to the pII promoter in KGN cells, which are derived from human granulosa cell tumors [12]. Furthermore, in the study of Pelusi et al. using SF-1 knockout (KO) mice, KO mice with markedly decreased SF-1 expression in granulosa cells, they found that these KO mice had hypoplastic ovaries that contained a decreased number of follicles and lacked corpora lutea. Moreover, they had blunted induction of plasma estradiol in response to gonadotropins, and they had reduced ovarian expression of anti-mullerian hormone, as well as decreased gonadotropin-induced ovarian expression of aromatase and cyclin D2 [13]. Thus, SF-1 is considered to be a master regulator of steroidogenesis, and it promotes estrogen biosynthesis through the regulation of P450 aromatase gene promoter PII in the ovarian follicle [14]. Recently, Miller et al. reported that the SF-1 gene exhibits frequent genetic (LOH/base substitution) and epigenetic (methylation) alterations in ovarian tumors [15]. Hu et al. conducted a meta-analysis that suggested that SF-1 may play an important role in ovarian cancer initiation and progression. In addition, they noted that SF-1 protein expression is not significantly different between benign and malignant ovarian tumors. SF-1 is also expressed in stromal cells and promotes angiogenesis [16]. Recently, Ferraz-de-Souza et al. reported angiopoietin 2 as a novel target of SF-1. This indicated a potential for the development of a new ovarian cancer treatment through SF-1 inducing angiogenesis [17].

In case 1, immunoreactivity for SF-1 was detected in the functioning stromal cells, which almost accorded with p450 aromatase expression, although there was some difference in intensity between p450 aromatase expression and SF-1 expression. In case 2, assessing the gene expression levels for *SF-1* and *CYP19A1* in the tumor by RT-qPCR showed that they were consistent with the results of immunohistochemical studies; both *SF-1* and *CYP19A1* genes were expressed in the tumor. Based on these facts, it is reasonable to suggest that the overexpression of SF-1 may promote estrogen biosynthesis through regulation of P450 aromatase expression in ovarian tumors with functioning stroma, and this in turn induces a high serum E_2_ level.

In addition, the E_2_ released from these ovarian mucinous adenocarcinomas has biological effects on the enlarged uterus and the incidence of abnormal vaginal bleeding or abnormally thick endometrium. The study of the radioanatomy of the uterus on MRI has indicated that the normal post-menopausal uterus has a relatively high intensity signal on T1 and T2-weighted sequences, close to that of fatty tissue. It also indicated that the size of the uterus (uterine height, uterine body length, and uterine cavity thickness) was significantly smaller than that of the premenopausal uterus. The threshold values to classify the uterine height, uterine body length, and uterine cavity thickness as normal on sagittal T2-weighted images were < 70 mm, < 50 mm, and <1 mm, respectively, for postmenopausal women [18, 19]. Based on these facts, the size of the uterus of Case 1 was clearly larger than that of a normal postmenopausal uterus.

There is strong in vitro and in vivo evidence that estrogens (and potentially other steroids) regulate ovarian carcinogenesis [20]. Furthermore, there has been increasing epidemiologic data, which come from individual participant datasets from 52 epidemiological studies involved in prospective studies (with last hormone therapy use extrapolated forwards for up to 4 years) in postmenopausal women. A meta-analysis showed that ovarian cancer risk was significantly greater in ever-users than in never-users of hormone therapy. However, regarding histopathological type, risk was definitely increased only for the two most common types, serous and endometrioid, but not mucinous and clear adenocarcinoma [21]. Sie at al. examined whether tumor expressions of the progesterone receptor (PR) and estrogen receptor (ER) were associated with subtype-specific survival. They found that PR and ER are prognostic biomarkers for only endometrioid and high-grade serous ovarian cancers, not for mucinous ovarian cancer [22]. This may be one of the reasons why serum estrogen levels in postmenopausal women have not been linked to biological behavior for ovarian mucinous adenocarcinoma. Chen et al. investigated the influence of sex hormone levels on tumor biology and patients’ outcomes in ovarian cancer. They concluded that there is no clear evidence of a prognosis marker or progression of elevated serum estradiol levels for common epithelial ovarian cancer including mucinous ovarian cancer [23]. Based on these findings, serum estrogen levels in postmenopausal women are not linked to the biological behavior of ovarian mucinous adenocarcinoma. Steroid hormone signaling in ovarian cancer is complex, yet little understood. Further investigations are needed.

## Conclusions

The present case showed that some elderly women with common epithelial tumors have an extremely high E_2_ level. Immunohistochemical and RT-qPCR evaluations showed that SF-1 may promote estrogen biosynthesis through regulation of P450 aromatase expression in ovarian tumors with functioning stroma, and that this in turn induces a high serum E_2_ level.

Further large-scale studies should provide additional insights into the mechanism whereby ovarian tumors, which contain functioning stroma with a high serum estrogen level and high expressions of P450 aromatase and SF-1, affect the prognosis and clinical outcomes of ovarian tumor patients. These studies have the potential to identify new biomarkers for anti-angiogenetic therapy and new treatments for some ovarian tumors.

## Consent

Written, informed consent was obtained from the patients for publication of these case reports and accompanying images. Copies of the written consents are available for review by the editor-in-chief of this journal.
